# Clustering and prediction of long-term functional recovery patterns in first-time stroke patients

**DOI:** 10.3389/fneur.2023.1130236

**Published:** 2023-03-08

**Authors:** Seyoung Shin, Won Hyuk Chang, Deog Young Kim, Jongmin Lee, Min Kyun Sohn, Min-Keun Song, Yong-Il Shin, Yang-Soo Lee, Min Cheol Joo, So Young Lee, Junhee Han, Jeonghoon Ahn, Gyung-Jae Oh, Young-Taek Kim, Kwangsu Kim, Yun-Hee Kim

**Affiliations:** ^1^Department of Physical and Rehabilitation Medicine, Center for Prevention and Rehabilitation, Heart Vascular Stroke Institute, Samsung Medical Center, Sungkyunkwan University School of Medicine, Seoul, Republic of Korea; ^2^Department and Research Institute of Rehabilitation Medicine, Yonsei University College of Medicine, Seoul, Republic of Korea; ^3^Department of Rehabilitation Medicine, Konkuk University School of Medicine, Seoul, Republic of Korea; ^4^Department of Rehabilitation Medicine, College of Medicine, Chungnam National University, Daejeon, Republic of Korea; ^5^Department of Physical and Rehabilitation Medicine, Chonnam National University Medical School, Gwangju, Republic of Korea; ^6^Department of Rehabilitation Medicine, Pusan National University School of Medicine, Pusan National University Yangsan Hospital, Yangsan-si, Republic of Korea; ^7^Department of Rehabilitation Medicine, School of Medicine, Kyungpook National University, Kyungpook National University Hospital, Daegu, Republic of Korea; ^8^Department of Rehabilitation Medicine, Wonkwang University School of Medicine, Iksan, Republic of Korea; ^9^Department of Rehabilitation Medicine, Jeju National University Hospital, Jeju National University School of Medicine, Jeju-si, Republic of Korea; ^10^Department of Statistics, Hallym University, Chuncheon-si, Republic of Korea; ^11^Department of Health Convergence, Ewha Womans University, Seoul, Republic of Korea; ^12^Department of Preventive Medicine, School of Medicine, Wonkwang University, Iksan, Republic of Korea; ^13^Department of Preventive Medicine, Chungnam National University Hospital, Daejeon, Republic of Korea; ^14^College of Computing, Sungkyunkwan University, Suwon-si, Republic of Korea; ^15^Department of Health Sciences and Technology, Department of Medical Device Management and Research, Department of Digital Health, Samsung Advanced Institute for Health Sciences & Technology (SAIHST), Sungkyunkwan University, Seoul, Republic of Korea

**Keywords:** stroke, functional recovery, artificial intelligence, machine learning, clustering, prediction

## Abstract

**Objectives:**

The purpose of this study was to cluster long-term multifaceted functional recovery patterns and to establish prediction models for functional outcome in first-time stroke patients using unsupervised machine learning.

**Methods:**

This study is an interim analysis of the dataset from the Korean Stroke Cohort for Functioning and Rehabilitation (KOSCO), a long-term, prospective, multicenter cohort study of first-time stroke patients. The KOSCO screened 10,636 first-time stroke patients admitted to nine representative hospitals in Korea during a three-year recruitment period, and 7,858 patients agreed to enroll. Early clinical and demographic features of stroke patients and six multifaceted functional assessment scores measured from 7 days to 24 months after stroke onset were used as input variables. K-means clustering analysis was performed, and prediction models were generated and validated using machine learning.

**Results:**

A total of 5,534 stroke patients (4,388 ischemic and 1,146 hemorrhagic; mean age 63·31 ± 12·86; 3,253 [58.78%] male) completed functional assessments 24 months after stroke onset. Through K-means clustering, ischemic stroke (IS) patients were clustered into five groups and hemorrhagic stroke (HS) patients into four groups. Each cluster had distinct clinical characteristics and functional recovery patterns. The final prediction models for IS and HS patients achieved relatively high prediction accuracies of 0.926 and 0.887, respectively.

**Conclusions:**

The longitudinal, multi-dimensional, functional assessment data of first-time stroke patients were successfully clustered, and the prediction models showed relatively good accuracies. Early identification and prediction of long-term functional outcomes will help clinicians develop customized treatment strategies.

## Introduction

Although early stroke management and rehabilitation protocols have improved during the past decade, stroke remains the most common cause of adult physical disability worldwide (1). The number of stroke survivors and the related overall global burden of stroke are both increasing (2). The ability to predict long-term recovery and prognosis of functional deficits after stroke is of interest. If clinicians could foresee the long-term functional recovery prospects for a certain patient, they could devise better treatment strategies.

Previous studies have attempted to develop algorithms to predict the prognosis for recovery after stroke (3). However, such algorithms considered only a single time point, making it difficult to establish overall recovery patterns. Douiri et al. (4) suggested creating decision curves to produce dynamic, time-dependent, multivariate, patient-specific predictive models that could overcome those limitations. Focusing on this attempt and the results of previous studies, it seems to be necessary to include the multifaceted functional outcomes for future prediction models. Because stroke patients often suffer from distinct motor, language, cognitive, and swallowing dysfunction, measuring only their ability to perform activities of daily living (ADL) is insufficient to classify patient-specific recovery patterns.

According to a previous study, lifestyle also is an important factor in stroke outcome (5). Therefore, when designing a prognosis prediction model for stroke patients, lifestyle factors need to be considered. The Korean Stroke Cohort for Functioning and Rehabilitation (KOSCO) (2) includes clinical characteristics; serial data of various functional domains; and lifestyle factors such as alcohol, smoking, and education level.

Therefore, in this study, we used a clustering analysis based on an unsupervised machine learning method that is suitable for classifying large, real-world KOSCO datasets containing longitudinal, multi-dimensional, functional assessments. Our primary aim in this study was to identify multifaceted, functional recovery patterns among first-time stroke patients using an unsupervised machine learning algorithm. Our secondary aim was to generate a prediction model for those recovery pattern clusters and examine the accuracy of the models.

## Methods

### Study populations

This study used data from the KOSCO study (6), a long-term, prospective, multicenter cohort study of residual disability and functional independence among Korean stroke patients following their first stroke episode.

**Figure 1:**
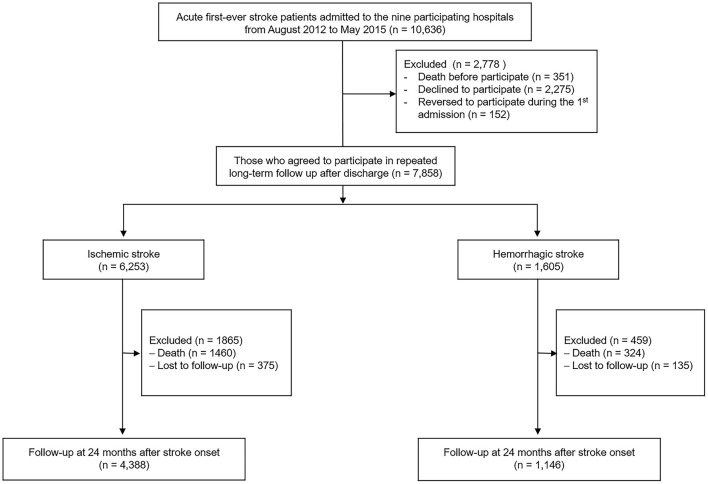
Flow chart of participant inclusion.

Between August 2012 and May 2015, the KOSCO study recruited 10,636 Korean patients. The inclusion criteria were (1) first-time acute IS or HS with a corresponding lesion on computed tomography or magnetic resonance imaging/angiography, (2) at least 19 years of age at stroke onset, and (3) onset of symptoms within 7 days prior to study enrollment. Patients with any of the following criteria were excluded: (1) transient ischemic attack, (2) history of previous stroke, and (3) traumatic intracerebral hemorrhage. Of the 10,636 first-time stroke patients (8,210 IS patients and 2,426 HS patients) admitted to nine representative hospitals in Korea during the recruitment period, 7,858 (6,253 IS patients and 1,605 HS patients) agreed to enroll after exclusion of patients who died or declined to participate. Among them, 5,534 patients (4,388 IS patients and 1,146 HS patients) who completed their follow-up assessments through 24 months after stroke onset were used in this analysis (Figure 1).

Written informed consent was obtained from all patients prior to inclusion, and the study protocol was approved by the ethics committees of the involved hospitals (Supplementary material).

### Measurements

#### Demographic and clinical characteristics

We considered the following demographic and clinical characteristics: sex, age, obesity (body mass index ≥ 26), education level (high: more than 9 years, low: <9 years), and stroke location (right, left, or both). Stroke severity was measured by the National Institutes of Health Stroke Scale (NIHSS) for 7 days after stroke onset for both IS and HS because the time from stroke onset to emergency department admission was different for each patient. A previous study showed that NIHSS is a reliable tool for clinical monitoring not only IS, but also HS patients (7). Combined condition- and age-related score (CCAS) according to Charlson Comorbidity Index, smoking, and history of alcohol consumption also was assessed. History of patient risk factors such as hypertension (systolic blood pressure > 160 mm Hg, diastolic blood pressure > 90 mm Hg, or history of hypertension or medical treatment), diabetes mellitus (DM; blood glucose level >126 mg/d or history of DM or medical treatment), hyperlipidemia (elevated low-density lipoprotein cholesterol level >160 mg/dL, elevated total cholesterol level > 240 mg/dL, or history of hy-perlipidemia or medical treatment), and atrial fibrillation (documented by standard electrocardiogram [ECG], long-term ECG, or history of atrial fibrillation or medical treatment) was assessed. Medical complications such as pneumonia and urinary tract infection during admission period were also included.

#### Functional assessments

The multifaceted functional assessments included six international tools: the Fugl-Meyer Assessment (FMA; range 0–100, higher score means higher motor function) (8) for motor function, the Functional Ambulation Classification (FAC; range 0–5, higher score means higher ambulatory function) (9) for mobility and gait, the Korean Mini-mental State Examination (K-MMSE; range 0–30, higher score means higher cognitive function) (10) for cognition, the short version of the Korean version of the Frenchay Aphasia Screening Test (short K-FAST; range 0–20, higher score means higher language function) (11) for language function, the American Speech-Language-Hearing Association's National Outcomes Measurement System (ASHA-NOMS; range 1–7, higher score means higher swallowing function) (12) for swallowing function, and the Korea Modified Barthel Index (K-MBI; range 0–100, higher score means higher activities of daily living performance independence) (13) for ADL function. Serial data from face-to-face functional assessments were gathered 7 days and 3, 6, 12, 18, and 24 months after stroke onset for all measures except K-MBI. K-MBI was not assessed at 7 days after stroke because most patients remained in the stroke unit for intensive care during the first week of admission.

The investigators of the KOSCO study were expert occupational therapists and underwent a standardized training program every 3 months to maintain inter-rater reliability.

### Clustering of functional recovery patterns

Clustering of functional recovery patterns was performed in first-time IS and HS stroke survivors. In order to select the most suitable clustering algorithm for the KOSCO dataset, we performed prior clustering using three well-known algorithms of K-means clustering (14), the Gaussian Mixture Model (15), and the Agglomerative clustering algorithm (15) and compared the functional scores. With these three algorithms, prior clustering was performed for cluster numbers 2–15, and the Silhouette Index (SI) (16) was estimated (Supplementary Figure 1). Among the three clustering algorithms, the K-means method was chosen for its higher SI for both IS and HS than the others.

The K-means clustering algorithm is one of the most popular unsupervised machine learning algorithms that partitions a dataset into a given number of clusters. The algorithm gathers each data point to the nearest centroid according to the number of clusters (17). To choose the optimal number of clusters that is not only able to explain the clinical features of patients, but also suitable for practical use, we used three functional scores: SI, Davies-Boulding Index (DBI) (18), and Calinski-Harabasz Index (CHI) (19). Cluster sizes from *k* = 2 to *k* = 15 were tested (Supplementary Figure 2). To avoid dividing into the highest, middle, and lowest groups, we considered only *k* >3. In IS, *k* = 5 showed tolerable functional scores (SI 0.47, DBI 1.43, and CHI 2487.07). In HS, *k* = 4 showed similar SI scores to that of IS (SI 0.42, DBI 1.36, CHI 875.67).

Finally, *K*-means clustering was performed with 100% of the dataset (4,388 IS and 1,146 HS patients). In this step, early clinical and demographic features of stroke patients and the repeated multifaceted functional assessment scores until 24 months were used as input variables. Missing or incomplete data were imputed using the *k*-nearest neighbor-5 (kNN-5) method (20). To confirm proper clustering, we visualized the clustered groups in low-dimensional images derived by t-Distributed Stochastic Neighbor Embedding (t-SNE) (21), which is widely used to convert high-dimensional data into a two- or three-dimensional map.

### Prediction model for long-term functional recovery

After clustering, we generated models that predict the cluster of new first-time stroke patients based on basic demographic data and functional scores from 7 days to 3 months after stroke onset. We used 70% of the dataset (3,071 IS and 802 HS patients) to generate the models and the remaining 30% (1,317 IS and 344 HS patients) for validation. Prediction models were simultaneously generated by eight machine learning algorithms: Light Gradient Boosting Machine (Light GBM) (22), extended version of Light GBM (Light GBM-XT) (22), Random Forest (RF) (23), CatBoost (CB) (24), extreme gradient boosting (XGBoost) (25), Weighted Ensemble (26), Neural Network (27), and Extra Trees (28). The performance metrics true positive (*TP*), true negative (*TN*), false positive (*FP*), and false negative (*FN*) were calculated. The mathematical expressions for F1 score, precision, and recall were as follows:


$$\begin{array}{l} \text{ Precision (PR) was given by: PR = }\frac{\text{TP}}{\text{TP + FP}}\\ \text{ Recall (RC) was given by: RC = }\frac{\text{TP}}{\text{TP + FN}}\\ {\text{F1 score was given by: F}}_{\beta }\text{ = }\frac{\text{(1 + }\beta^{\text{2}}\text{ ) · PR ·RC}}{\text{(}\beta^{\text{2}}\text{(PR + RC) )}} \end{array}$$

where β represents the weighted value between precision and recall. In this case, β = 1.

The accuracy of the overall prediction model for each IS and HS was calculated as follows:


$$\begin{array}{l} \text{Accuracy}=\frac{\text{TP}+\text{TN}}{\text{TP}+\text{TN}+\text{FP}+\text{FN}} \end{array}$$

### Computational details

Descriptive statistical analyses and within-group comparison were implemented in R (version 4.0.3). The independent *t*-test was used for comparison of continuous variables, and the chi-square test was used for comparison of categorical variables between IS and HS. The level of significance was set as two-sided *p* < 0.05. All machine learning steps of data analysis, preprocessing of model training, and visualization were performed using open source libraries in Python (version 3.9.0). Pandas (version 1.5.2) was used for data analysis and preprocessing, and scikit-learn (version 1.2.0) was used to impute missing values and to establish the clustering model. Visualization was conducted through plotly (version 5.9.0) and matplitlib (version 3.6.2). Model predictions were performed using Autogluon (version 0.6.2).

## Results

### Patient characteristics

The demographic and clinical characteristics of the participants are provided in Table 1. Of the 5,534 patients who underwent functional assessment 24 months after stroke onset, 4,388 were IS (2,700 males) and 1,146 were HS (553 males) patients. The mean age (standard deviation, SD) of IS patients was 64.8 (SD, 12.4) years, and their mean NIHSS score at 7 days was 3.5 (SD, 5.1). There were significant differences between IS and HS patients in demographic and clinical characteristics except obesity and alcohol history *(p* < 0.001).

**Table 1:** Demographic and clinical characteristics of the participants.

	**All stroke patients (*n* = 5,534)**	**Ischemic stroke (*n* = 4,388)**	**Hemorrhagic stroke (*n* = 1,146)**	***P-* value^a^**
Age, years	63.31 ± 12.86 (19–98)	64.79 ± 12.38 (19–98)	57.64 ± 13.10 (20–92)	<0.001^***^
Sex, male	3,253 (58.78%)	2,700 (61.53%)	553 (48.26%)	<0.001^***^
Education				
<9 years	3,776 (68.23%)	2,915 (66.43%)	861 (75.13%)	<0.001^***^
≥9 years	1,758 (31.77%)	1,473 (33.57%)	285 (24.87%)	<0.001^***^
Stroke location				
Right	2,297 (41.51%)	1,975 (45.01%)	322 (28.10%)	<0.001^***^
Left	2,431 (43.93%)	2,125 (48.43%)	306 (26.70%)	<0.001^***^
Both	806 (14.56%)	288 (6.56%)	518 (45.20%)	<0.001^***^
Risk factors				
Hypertension, yes	2,937 (53.07%)	2,446 (55.74%)	491 (42.84%)	<0.001^***^
Diabetes mellitus, yes	1,160 (20.96%)	1,048 (23.88%)	112 (9.77%)	<0.001^***^
Atrial fibrillation, yes	388 (7.01%)	371 (8.45%)	17 (1.48%)	<0.001^***^
Hyperlipidemia, yes	510 (9.22%)	460 (10.48%)	50 (4.36%)	<0.001^***^
Obesity (BMI ≥ 26), yes	615 (11.11%)	502 (11.44%)	113 (9.86%)	0.14
CCAS ≥ 6, yes	2,319 (41.90%)	1,989 (45.33%)	330 (28.80%)	<0.001^***^
Smoking, yes	1,505 (27.20%)	1,244 (28.35%)	261 (22.77%)	<0.001^***^
Alcohol, yes	2,228 (40.26%)	1,751 (39.90%)	477 (41.62%)	0.31
Complications				
Pneumonia, yes	137 (2.48%)	78 (1.78%)	59 (5.15%)	<0.001^***^
Urinary tract infection, yes	135 (2.44%)	80 (1.82%)	55 (4.80%)	<0.001^***^
NIHSS at 7 days	4.05 ± 6.23 (0–42)	3.53 ± 5.11 (0–42)	6.71 ± 8.85 (0–42)	<0.001^***^
Functional assessments at 7 days				
FMA	78.77 ± 32.41 (0–100)	81.89 ± 29.73 (0–100)	66.84 ± 38.83 (0–100)	<0.001^***^
FAC	2.89 ± 1.91 (0–5)	3.17 ± 1.80 (0–5)	1.82 ± 1.95 (0–5)	<0.001^***^
K-MMSE	21.61 ± 8.79 (0–30)	22.64 ± 7.98 (0–30)	17.65 ± 10.45 (0–30)	<0.001^***^
Short K-FAST	13.01 ± 6.35 (0–20)	13.63 ± 5.92 (0–20)	10.65 ± 7.30 (0–20)	<0.001^***^
ASHA-NOMS	5.92 ± 1.95 (1–7)	6.13 ± 1.76 (1–7)	5.11 ± 2.38 (1–7)	<0.001^***^
Functional assessments at 3 months				
K-MBI	86.37 ± 25.35 (0–100)	87.89 ± 23.55 (0–100)	80.58 ± 30.63 (0–100)	<0.001^***^

Values are mean ± standard deviation (range) or number (%).

^**a**^The independent t-test was used for continuous variables and the chi-square test was used for categorical variables between ischemic and hemorrhagic stroke types.

^***^p <0.001.

BMI, Body mass index; CCAS, Combined condition- and age-related score; NIHSS, National Institutes of Health Stroke Scale; FMA, Fugl-Meyer Assessment; FAC, Functional Ambulatory Category; K-MMSE, Korean Mini-Mental State Examination; Short K-FAST, Short Korean-language Frenchay Aphasia Screening Test; ASHA-NOMS, American Speech-Language-Hearing Association National Outcome Measurement System Swallowing Scale; K-MBI, Korean Modified Barthel Index.

### Clustering of long-term functional outcomes in survivors of first-time stroke

**Figure 2:**
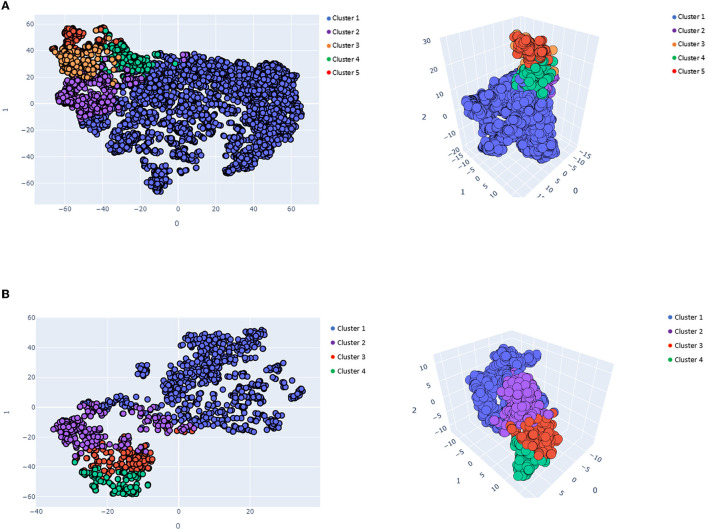
Visualized *K*-means clusters results in 2- and 3-dimensional spaces. **(A)** Visualized ischemic *K*-means clusters results (*k* = 5), **(B)** Visualized hemorrhagic *K*-means cluster results (*k* = 4).

Regarding the *K*-means clustering algorithm, the optimal number of clusters was five for IS patients and four for HS patients. In both the IS and HS groups, the clusters differed in mean age, initial stroke severity as measured by NIHSS scores, complications, and comorbidities. Figure 2 visualizes IS (Figure 2A) and HS (Figure 2B) *K*-means clustering using t-SNE.

**Figure 3:**
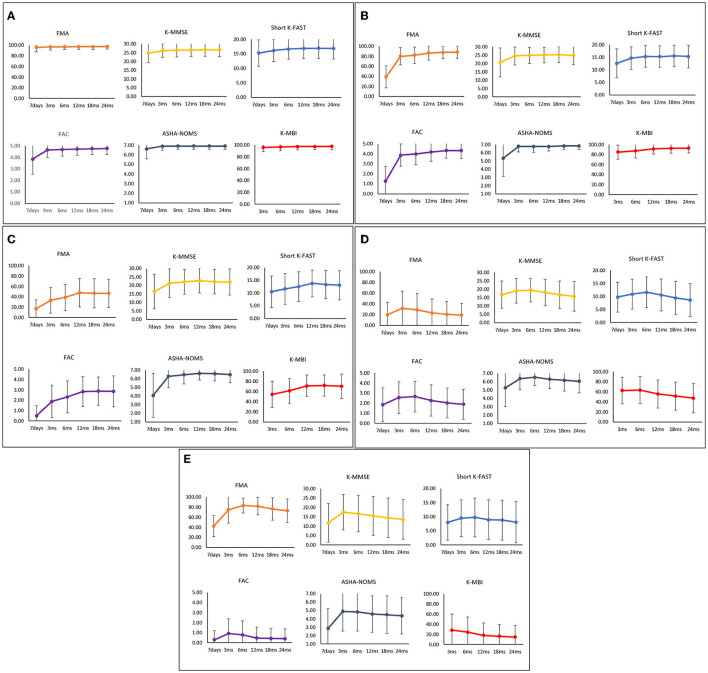
Functional recovery patterns of clusters of ischemic stroke patients until 24 months after onset. **(A)** Cluster 1 (*n* = 3,346); minimal functional deficit in all domains, **(B)** Cluster 2 (*n* = 405); rapid improvement of motor and ambulatory functions during the subacute phase, **(C)** Cluster 3 (*n* = 232); significant motor and ambulatory dysfunctions with continuous improvement, **(D)** Cluster 4 (*n* = 204); moderate dysfunctions with late phase decrement, **(E)** Cluster 5 (*n* = 201); severe dysfunctions in all domains. FMA, Fugl-Meyer Assessment; FAC, Functional Ambulatory Category; K-MMSE, Korean Mini-Mental State Examination; Short K-FAST, Short Korean version of the Frenchay Aphasia Screening Test; AHSA-NOMS, American Speech-Language-Hearing Association National Outcome Measurement System Swallowing Scale; K-MBI, Korean modified Barthel Index.

The functional recovery characteristics of the final five clusters of IS patients are presented in Supplementary Table 1 and Figure 3. Cluster 1, which contained 3,346 patients (60.46%), was characterized by a mean age of 63.53 years (SD, 12.25) and a low 7-day stroke severity with a mean of 1.44 (SD, 1.81). These patients showed minimal deficits in every functional domain. Cluster 2, comprising 405 patients (7.32%), was characterized by a mean age similar to cluster 1 (mean [SD]; 64.47 years [12.12]) and moderate initial severity (7.07 [5.24]). This cluster showed low motor and ambulatory functions at onset but rapidly improved during the subacute phase. Cluster 3, comprising 232 patients (4.19%), was characterized by a mean age of 64.46 years (SD, 10.87), with a low but moderately severe NIHSS score of 11.88 (SD, 5.82). This group showed significant motor and ambulatory dysfunction compared to cognitive and language functioning; however, they showed continuous improvement during the 24-month study period. In contrast, ADL and cognitive and language functions showed little decline after 12 months. Cluster 4, which contained 204 patients (3.69%), was characterized by a relatively older mean age of 76.24 years (SD, 8.44) and a 7-day NIHSS score of 5.35 (SD, 4.74). All functional domains showed dysfunction, especially the motor and ambulatory domains, which showed little improvement during the first 6 months and then decreased later. Cluster 5, which contained 201 patients (3.63%), was characterized by relatively older age, with a mean of 75.10 years (SD, 8.92), and a higher mean initial NIHSS score of 15.90 (SD, 8.07). This cluster showed the worst functional recovery over all six functional domains.

**Figure 4:**
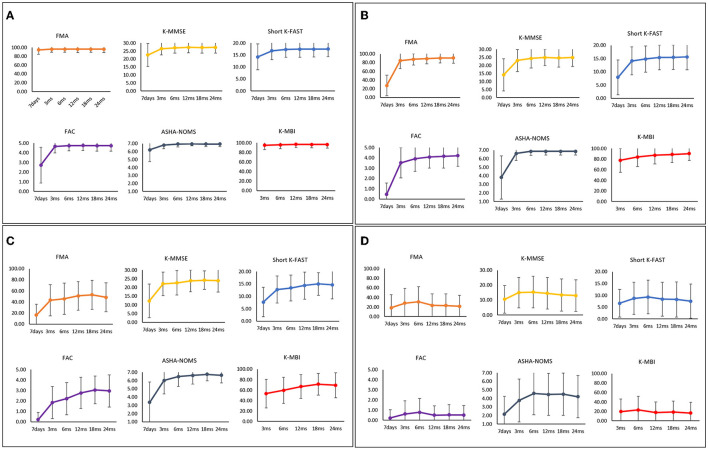
Functional recovery patterns of clusters of hemorrhagic stroke patients until 24 months after onset. **(A)** Cluster 1 (*n* = 710); minimal functional deficit in all domains, **(B)** Cluster 2 (*n* = 208); early rapid recovery of all functions, **(C)** Cluster 3 (*n* = 128); significant improvement in all domains over 24 months after stroke with lower motor and ambulatory function, **(D)** Cluster 4 (*n* = 100); severe dysfunctions in all domains. FMA, Fugl-Meyer Assessment; FAC, Functional Ambulatory Category; K-MMSE, Korean Mini-Mental State Examination; Short K-FAST, Short Korean version of the Frenchay Aphasia Screening Test; AHSA-NOMS, American Speech-Language-Hearing Association National Outcome Measurement System Swallowing Scale; K-MBI, Korean modified Barthel Index.

The final four clusters of HS patients and their functional recovery characteristics are presented in Supplementary Table 1 and Figure 4. The 710 patients (61.95%) in cluster 1 were characterized by young age (55.99 years [12.64]) and mild initial severity (1.61 [2.61]), similar to IS cluster 1. Cluster 2, which contained 208 patients (18.15%), was characterized by a mean age of 59.02 years (SD, 13.40) and a 7-day NIHSS of 12.51 (SD, 8.68). These patients had low scores in motor, cognitive, language, ambulatory, and swallowing functions at 7 days after stroke onset, but they recovered rapidly, especially in the motor and ambulatory domains. Cluster 3, comprising 128 patients (11.17%), was characterized by a mean age of 57.56 years (SD, 12.66) and a 7-day NIHSS of 15.44 (SD, 7.71). The average age was younger than in cluster 2, but the initial stroke severity was slightly higher. All functional domains showed low scores at 7 days after stroke and improved significantly during the first 3 months. Those patterns were similar to those of cluster 2. However, motor and ambulatory functions were much lower in cluster 3. Cluster 4, which contained 100 patients (8.73%), was characterized by older age (66.52 years [12.44]) and the highest initial severity (19.59 [9.92]). All domains showed the lowest initial scores with a slight improvement in the first 3 months; however, these patients showed little improvement or even worse performance over time.

### Predicting recovery patterns after first-time stroke

The predictive model was developed to predict the recovery pattern for up to 24 months with only patient information up to the subacute phase of stroke. The input variables were demographic features and functional scores assessed 7 days and 3 months after stroke onset. The accuracy, F1 score, precision, and recall scores of the final IS cluster (*k* = 5) are demonstrated in Table 2. Among the eight machine learning models, CatBoost and Light GBM-XT showed the best performance (accuracy, 0.926 and 0.925, respectively). All other models showed accuracies higher than 0.90 for IS (XGBoost, 0.920; RF, 0.919; Light GBM, 0.917; Weighted Ensemble, 0.917; Neural Net, 0.912; Extra trees, 0.912).

**Table 2:** Performance scores of the prediction models.

**Ischemic stroke**					**Hemorrhagic stroke**				
**Model**	**Accuracy**	**F1** ^**a**^	**Precision** ^**a**^	**Recall** ^**a**^	**Model**	**Accuracy**	**F1** ^**a**^	**Precision** ^**a**^	**Recall** ^**a**^
CatBoost	0.926	0.751	0.780	0.737	CatBoost	0.887	0.778	0.796	0.771
Light GBM-XT	0.925	0.747	0.789	0.731	Light GBM-XT	0.887	0.793	0.802	0.789
XGBoost	0.920	0.728	0.809	0.708	Light GBM	0.887	0.786	0.795	0.785
Random Forest	0.919	0.722	0.764	0.711	XGBoost	0.883	0.779	0.837	0.765
Light GBM	0.917	0.725	0.764	0.718	Weighted Ensemble	0.883	0.775	0.799	0.765
Weighted Ensemble	0.917	0.725	0.764	0.718	Random Forest	0.883	0.77	0.786	0.765
Neural Network	0.912	0.714	0.809	0.699	Neural Network	0.870	0.749	0.750	0.749
Extra Trees	0.912	0.702	0.757	0.683	Extra Trees	0.861	0.735	0.746	0.732

^a^Values are presented as mean values for every cluster.

Light GBM, Light Gradient Boosting Machine; Light GBM-XT, extended version of Light GBM; XGBoost, extreme gradient boosting.

The performance scores of the final HS cluster (*k* = 4) are described in Table 2. Both CatBoost and Light GBM-XT showed the highest accuracy of 0.887, and the Extra trees model showed the lowest accuracy of 0.861. All other models showed accuracies higher than 0.85 for HS (Light GBM, 0.887; XGBoost, 0.883; Weighted Ensemble, 0.883; RF, 0.883; Neural Net, 0.870; Extra trees, 0.861).

The detailed parameters of CatBoost and Light GBM-XT models, which showed the highest accuracies in this study, are described in the Supplementary material.

## Discussion

In this study, we used an unsupervised machine learning algorithm to extract clusters of long-term, multifaceted functional recovery patterns in first-time stroke patients. After identifying the most suitable algorithm and number of clusters for our data, we identified five distinct IS clusters and four HS clusters based on clinical and demographic features. All prediction models for the IS and HS clusters achieved accuracies of 0.90 and 0.85, respectively, when using demographic, 7-day, and 3-month functional data after stroke. Among the models evaluated, CatBoost and Light GBM-XT showed the best performance in both IS and HS.

Recently, machine learning has played an increasing role in medical research. The number of publications about machine learning in the medical field is increasing annually. PubMed showed only 370 articles using machine learning in 2007; however, the number increased to 3,978 articles in 2017 (29). Machine learning has advantages in personalized medicine, handling large data sets, and design of prediction models. Indeed, multiple studies have attempted to predict prognoses after stroke using methods that showed relatively acceptable levels of accuracy (3, 30, 31). Among them, the Predicting Recovery Potential (PREP) (32) and Time to Walking Independently After Stroke (TWIST) (33) algorithms used decision trees, which is a machine learning method, to predict upper limb or walking abilities. The PREP algorithm for prognosis of upper limb motor recovery had a positive predictive power of 88%, specificity of 88%, and sensitivity of 73%. The TWIST algorithm for prognosis of independent gait at 3 months after stroke showed prediction accuracy for 95% of patients. Scrutinio et al. (34) compared three tree-based machine learning algorithms to predict whether a patient who suffered a severe stroke would be dead or alive 3 years later. The machine learning model that showed the highest performance score had an area under the curve (AUC) of 0.928 and an accuracy of 86.1%. Another recent study used five types of machine learning algorithms to predict favorable outcomes (modified Rankin Scale 0 or 1) for acute IS patients at 3 months (35) and revealed that all five algorithms had an AUC >0.8. All those studies suggest the possibilities and usefulness of machine learning in clinical medicine.

The KOSCO data are characterized by multi-time point longitudinal and multivariate assessments. A previous study using KOSCO data suggested that long-term functional recovery patterns varied by patient baseline characteristics (36). This indicates that it would be difficult to predict prognosis using only fragmentary information. Moreover, our primary aim was not just to predict a binary classification at a specific time point, but to predict functional recovery patterns of six domains over 24 months.

The KOSCO data indicated that 60.46% of IS patients (cluster 1) and 61.95% of HS patients (cluster 1) showed minimal dysfunction after stroke. Another 11.51% of IS patients (clusters 2 and 3) and 29.32% of HS patients (clusters 2 and 3) showed significant improvement over 24 months, reaching near full or significant recovery from their dysfunction, though some exceptions showed unsatisfactory recovery. In addition, 201 (4.58%) IS patients (cluster 5) and 100 (8.73%) HS patients (cluster 4) showed severe dysfunction from the onset of stroke. Another 3.69% of IS patients (cluster 4) showed a decline in functional outcomes during the late phase of follow-up. Overall, clusters with older patients presented worse outcomes than those of younger patients. The reason for the larger number of clusters of poor prognoses in IS (cluster 4 and 5) than HS (cluster 4) likely is not only the larger number of patients, but also their older mean age.

For both stroke types, CatBoost and Light GBM-XT were the most suitable of the eight machine learning algorithm prediction models for the KOSCO data. Whereas previous studies targeted only one or two time points and used few types of functional outcomes in their predictions, our study provides stronger evidence. We used six functional assessments to characterize motor, mobility, cognitive, language, and swallowing functions and ADL independence at five to six time points. Therefore, our study can describe patients in a more detailed and accurate manner than previous studies and shows time-course changes. Although we analyzed high-dimensional clinical data from a large, long-term cohort of patients, the accuracy of our best prediction model was 0.926 for IS and 0.887 for HS.

## Limitations and conclusions

This study has some limitations. First, because the KOSCO dataset contains long-term repetitive assessment data, some data were missing and were handled by statistical methods. First, because the KOSCO dataset contains long-term repetitive assessment data, some data were missing. All subjects included in this study were followed for up to 24 months after stroke, but some cases had missing data at some time point. In such cases, we imputed data using the k-NN5 method as described in the Method section. Second, we analyzed data only for those who survived at 24 months after stroke. Therefore, the subjects of this study may not represent all stroke patients in Korea. Finally, imaging biomarkers such as dynamic nomogram (37) or diffusion tensor image and functional MRI (38) also are useful predictors. However, the KOSCO study did not include such imaging biomarkers because there were limitations in the study design as a multicenter national study with a large number of subjects and many time points assessment.

Despite the above limitations, this study successfully clustered long-term functional recovery patterns in IS and HS patients using machine learning. Machine learning algorithms are increasing in efficacy and overcoming their limitations. Early identification and accurate prediction of long-term functional outcomes using machine learning will help clinicians to develop customized management strategies for stroke patients.

## Data availability statement

The data that support the findings of this study are available from the corresponding author, upon reasonable request.

## Ethics statement

The studies involving human participants were reviewed and approved by Samsung Medical Center, 2012-06-016 Severance Hospital, 4-2012-0341 Konkuk University Medical Center, 1180-01-700 Chungnam National University Hospital, 2012-06-011 Chonnam National University Hospital, CNUH-2012-127 Pusan National University Yangsan Hospital, 05-2012-057 Kyungpook National University Hospital, 2013-03-029 Wonkwang University Hospital, and 1515 Jeju National University Hospital, 2013-02-001. The patients/participants provided their written informed consent to participate in this study.

## Author contributions

Y-HK had full access to all the data in the study and takes responsibility for the integrity of the data and the accuracy of the data analysis. Conceptualization, project administration, and funding acquisition: Y-HK. Data curation and Investigation: Y-HK, SS, WHC, MKS, JL, DYK, Y-IS, G-JO, Y-SL, MCJ, SL, M-KS, JH, JA, and Y-TK. Methodology, writing—review, and editing: Y-HK, SS, WHC, MKS, JL, DYK, Y-IS, G-JO, Y-SL, MCJ, SL, M-KS, JH, JA, Y-TK, and KK. Formal analysis: SS, JH, and KK. Writing—original draft: SS. Resources: Y-HK, WHC, MKS, JL, DYK, Y-IS, G-JO, Y-SL, MCJ, SL, M-KS, JH, JA, and Y-TK. Supervision: Y-HK, MKS, JJ, DYK, Y-IS, G-JO, Y-SL, MCJ, SL, M-KS, JH, JA, Y-TK, and KK. All authors have read and approved the final manuscript and revised and approved the manuscript.
