# Supplementary material for: Clustering and prediction of long-term functional recovery patterns in first-time stroke patients
Source: Front Neurol. 2023 Mar 8;14:1130236. doi: 10.3389/fneur.2023.1130236 (PMC10031095; doi:10.3389/fneur.2023.1130236)
Supplement: Supplementary file 1 [file Data_Sheet_1.docx]

**Supplementary Methods**

**1. Ethics committees of participating hospitals**

Samsung Medical Center, 2012-06-016

Severance Hospital, 4-2012-0341

Konkuk University Medical Center, 1180-01-700

Chungnam National University Hospital, 2012-06-011

Chonnam National University Hospital, CNUH-2012-127

Pusan National University Yangsan Hospital, 05-2012-057

Kyungpook National University Hospital, 2013-03-029

Wonkwang University Hospital, 1515

Jeju National University Hospital, 2013-02-001

**2. Detailed parameters of prediction models with the highest accuracy**

CatBoost Model for Ischemic Stroke: {'learning_rate': 0·11259896076097305, 'random_seed': 0, 'depth': 5, 'l2_leaf_reg': 3·3713784729000733}

Light GBM-XT Model for Ischemic Stroke: {'extra_trees': True, 'learning_rate': 0·049883446878335284, 'feature_fraction': 0·9618129346960314, 'min_data_in_leaf': 52, 'num_leaves': 52}

CatBoost Model for Hemorrhagic Stroke: {'learning_rate': 0·014984363280426882, 'random_seed': 0, 'depth': 6, 'l2_leaf_reg': 2·5375268291707993}

Light GBM-XT Model for Hemorrhagic Stroke: {'extra_trees': True, 'learning_rate': 0·05, 'feature_fraction': 1·0, 'min_data_in_leaf': 20, 'num_leaves': 31}

**Supplementary Figure 1**

Silhouette index of the three different clustering algorithms according to the number of clusters. **(A)** Ischemic stroke. **(B)** Hemorrhagic stroke.

**
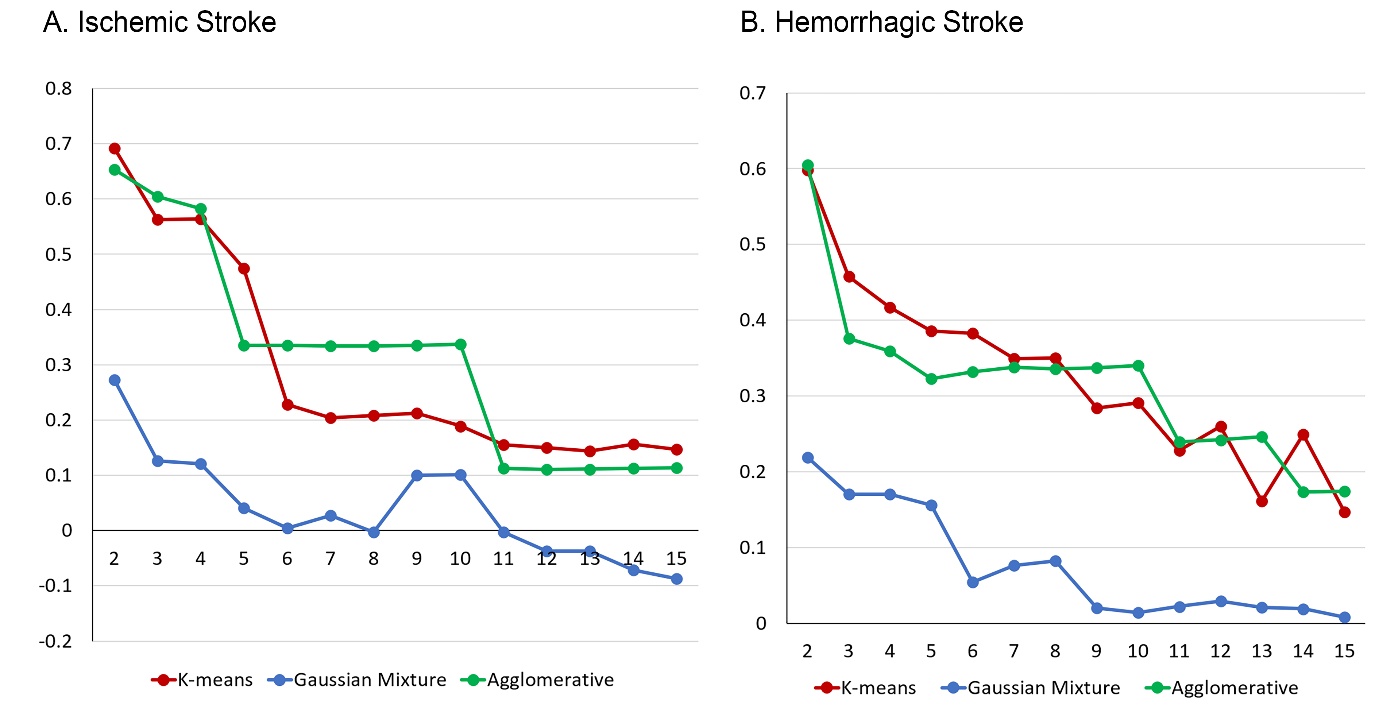
**

**Supplementary Figure 2**

Functional scores of the K-means clustering according to the number of clusters. **(A)** Ischemic stroke. **(B)** Hemorrhagic stroke

**
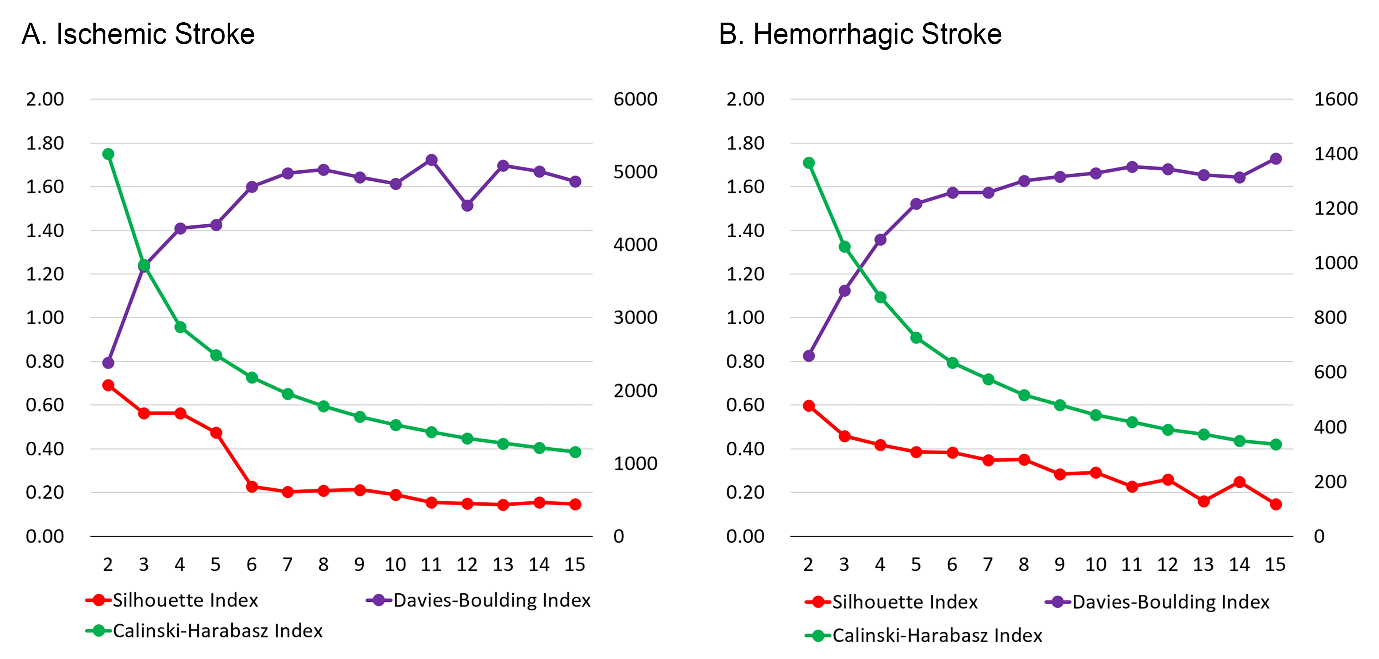
**

**Supplementary Table 1** Key defining features of the clinical profiles of ischemic and hemorrhagic stroke patients

| **Ischemic stroke clusters** | | | | **Hemorrhagic stroke clusters** | | | |
| --- | --- | --- | --- | --- | --- | --- | --- |
| **Cluster No.** | **N (%)** | **Mean age (years)** | **Cluster profile** | **Cluster No.** | **N (%)** | **Mean age (years)** | **Cluster profile** |
| **1** | 3,346 (76.25%) | 63.53 | Minimal functional deficit in all domains | **1** | 710 (61.95%) | 55.99 | Minimal functional deficit in all domains |
| **2** | 405  (9.23%) | 64.47 | Rapid improvement of motor and ambulatory function during the subacute phase | **2** | 208 (18.15%) | 59.02 | Early rapid recovery of all functions |
| **3** | 232 (5.29%) | 64.46 | Significant motor and ambulatory dysfunction with continuous improvement | **3** | 128 (11.17%) | 57.56 | Significant improvement in all domains over 24 months after stroke with lower motor and ambulatory function |
| **4** | 204  (4.65%) | 76.24 | Moderate dysfunction with late phase decrement | **4** | 100 (8.73%) | 66.52 | Severe dysfunction in all domains |
| **5** | 201 (4.58%) | 75.10 | Severe dysfunction in all domains |  |  |  |  |
